# Graduate Study on the Continent—Austria

**Published:** 1909-02-06

**Authors:** 


### GRADUATE STUDY ON THE CONTINENT—AUSTRIA.

### VII.—INNSBRUCK AND BUDAPEST.

To tourists Innsbruck is sufficiently well known; to post-graduates its attractions are not so thoroughly familiar. With its attractions as a "sojourn centre" and as a base for excursions we need not here occupy ourselves; suffice it to say that they compare favourably with those of Graz and Prague. As a centre for medical study it needs a slightly longer comment.

Innsbruck is a small centre, comparable to Göttingen or Marburg in Germany. It has the advantages which we have already pointed out as belonging exclusively to such small centres, but it has also some disadvantages which are peculiar to itself. Thus it is a large tourist base, with all the distractions, to foreign graduates at least, which such a busy centre brings. The winter term is long, while the summer term is unusually short. Living is comparatively expensive, though good rooms can be obtained at an average monthly rental of 30 kronen, exclusive of extras. Pensions and hotels are good, but in point of price compare unfavourably with other Austrian centres. The hospitals are small and the clinical material available is not very large or varied, as Innsbruck does not tap a large district owing to the vicinity of other large centres such as Munich and Prague. The university is the youngest in Austria, and its medical faculty is a small one. The following courses are given by the Docenten:—Clinical practical microscopy (Dr. Posselt), practical and theoretical parasitology (Dr. von Hibler), diagnosis and treatment of venereal diseases (Dr. Rusch), topographical anatomy (Dr. Greill), immunity (Dr. von Wundscheim), principles of serum treatment (Dr. Ballner). There is a small university library. The matriculation fee is 9 kronen. Lectures are paid for at various rates; none are specially limited to graduate practitioners.

Vacation courses are given here in the autumn, but have so far been badly attended. They are practical and theoretical, and the demonstrations are given by the professors and docenten. Application as to details should be made to the Dean of the Medical Faculty, University of Innsbruck. As the courses are limited, early application is desirable. We cannot, however, recommend the graduate student to devote much of his time to this centre. It is, of course, worth while to visit the hospital and to see methods here as elsewhere, but the lectures are not specially attractive to graduate students, and the mateiial available is limited. As a centre for study Innsbruck cannot honestly be compared with its other Austrian rivals. For all that, it is a pleasant centre at which to spend a few weeks and to occupy oneself as a free-lance, attending the lectures as quest-auditor and visiting the various clinics and polyclinics. More than that it does not hold out to the graduate student.

#### Budapest.

With Budapest the case is different, though here the student will find that a knowledge of German alone does not carry him very far. We may therefore eliminate formal lectures. The university of Budapest ranks among the largest and most important in Europe, and is only surpassed, so far as numerical strength of its matriculates is concerned, by the universities of Naples, Paris, Berlin, and Vienna. So far as university lectures are concerned, however, the English graduate will not be able to profit much by them, as they are given exclusively in Hungarian—a language which for purely professional purposes it is hardly worth while to learn.

In the fine hospitals the free-lance student will find much to interest him. Living at Budapest is comparatively cheap, and there is more opportunity for reference work than at Innsbruck or Prague, owing to the excellence of the libraries. Specially worth visiting are the out-patient departments of the medical and surgical clinics, the smaller special hospitals, of which we may here instance the Stephanie Children's Hospital, the Orthopædic Institute, and the small but very representative pathological collections. As a city Budapest surpasses Vienna by far in point of interest. Here the East and the West mingle, and from the purely clinical aspect this is of value to the graduate. Thus in parasitology, infectious diseases, diseases of occupation, and certain tropical or semi-tropical affections there is abundance of material. The hospitals are large and good. and they draw their patients not only from all parts of Hungary but from neighbouring countries such as Servia, Montenegro, Dalmatia, Turkey, Roumania, and Russia.

To Budapest, too, must belong the credit of standing in the front rank of Austrian centres at which vacation courses for practitioners are held. These courses have now been held for many years, and have received some State support, while they have been very popular with local practitioners. Courses are given during the summer and winter vacations in all subjects. Each member pays a course subscription of 20 kronen to cover secretarial and other expenses, and receives a card which admits him to all courses, practical as well as theoretical. These courses are under the auspices of a local committee on which the medical faculty of the university is fully represented, and are given in Hungarian. For particulars graduates should apply to the chairman of this committee, Professor Emil von Grösz, or to the secretary, Dr. Rudolf Temésvary, Schriftführer Arztliche Ferialkursen, Budapest.

#### Dental Post-graduate Study in Austria.

In all the Austrian centres the dental graduate will find opportunities for practical work and theoretical study. At every university there is a professor of dentistry and a dental institute. The latter is usually small, and only in Vienna can it be said to compare with the fine dental hospitals at Berlin and Munich. Lectures are given during the winter and summer terms, but there is no such special catering for the graduate student in dentistry as exists in Germany.

Austria is as yet an untrodden field so far as the English-speaking graduate is concerned. For one must not lose sight of the fact that Vienna does not constitute Austria. It would be as foolish for the graduate to spend his time at Berlin only and to say that he has learned German methods and German work as it is for him to devote himself entirely to Vienna and come away with the consciousness that he has nothing further to learn from Austrian hospitals. That is, however, a plan which is unfortunately pursued by many English-speaking graduates. In these articles we have tried to point out the claims which other centres have, and we would urge graduates to visit at least Prague and Graz as well as Vienna. Both these centres are thoroughly good, though they need "exploitation" in a clinical sense. The capital needs no advertisement; it has already gained as much as it deserves, and it will always secure a large amount of attention from all foreign graduates because of its reputation and because of the merits of its individual clinical teachers.

If one may summarise the advantages which Austria offers for graduate study, and compare these with the attractions of German centres, they lie chiefly in the concentration of the material and the possibilities for practical clinical work. There can be no doubt that graduate teaching, so far as course teaching is concerned, is better in Germany, where it is more thoroughly practical, less elementary, and where graduate courses are such truly and not mixed classes. In Germany also there is, perhaps, more opportunity for literary work, as the libraries are all good, even at the small centres, whereas in Austria they leave much to be desired. But no German centre can compare with the main Austrian cities for concentration of material. In Berlin, for instance, there is an enormous amount of clinical material, but it is broken up between a dozen or more hospitals and clinics, while in the Austrian centres, which have fewer hospitals than the population requires, all this material, equally rich and equally large, is concentrated in one or two chief institutions. For these reasons no graduate who wishes to study on the Continent should neglect to visit at least one Austrian centre.

